# Genome-Wide Differential Methylation Profiles from Two Terpene-Rich Medicinal Plant Extracts Administered in Osteoarthritis Rats

**DOI:** 10.3390/plants10061132

**Published:** 2021-06-02

**Authors:** Younhee Shin, Sathiyamoorthy Subramaniyam, Jin-Mi Chun, Ji-Hyeon Jeon, Ji-Man Hong, Hojin Jung, Boseok Seong, Chul Kim

**Affiliations:** 1 Research and Development Center, Insilicogen Inc., Yongin-si 16954, Korea; yhshin@insilicogen.com (Y.S.); moorthy@insilicogen.com (S.S.); jhjeon@insilicogen.com (J.-H.J.); jmhong@insilicogen.com (J.-M.H.); hjjung@insilicogen.com (H.J.); 2 Herbal Medicine Resources Research Center, Korea Institute of Oriental Medicine, 111 Geonjae-ro, Naju-si 58245, Korea; jmchun@kiom.re.kr; 3 Department of Biological Sciences, Sungkyunkwan University, Suwon 16419, Korea; 4 Future Medicine Division, Korea Institute of Oriental Medicine, 1672 Yuseong-daero, Yuseong-gu, Daejeon 34054, Korea; sungbosal@kiom.re.kr

**Keywords:** *Dipsacus asperoides*, osteoarthritis, *Phlomis umbrosa*, iridoid, triterpenoid, rats

## Abstract

Extracts from the plants *Phlomis umbrosa* and *Dipsacus asperoides*—which are widely used in Korean and Chinese traditional medicine to treat osteoarthritis and other bone diseases—were used to treat experimental osteoarthritis (OA) rats. Genome-wide differential methylation regions (DMRs) of these medicinal-plant-treated rats were profiled as therapeutic evidence associated with traditional medicine, and they need to be investigated further using detailed molecular research to extrapolate traditional practices to modern medicine. In total, 49 protein-encoding genes whose expression is differentially regulated during disease progression and recovery have been discovered via systematic bioinformatic analysis and have been approved/proposed as druggable targets for various bone diseases by the US food and drug administration. Genes encoding proteins involved in the PI3K/AKT pathway were found to be enriched, likely as this pathway plays a crucial role during OA progression as well as during the recovery process after treatment with the aforementioned plant extracts. The four sub-networks of PI3K/AKT were highly regulated by these plant extracts. Overall, 29 genes were seen in level 2 (51–75%) DMRs and were correlated highly with OA pathogenesis. Here, we propose that these genes could serve as targets to study OA; moreover, the iridoid and triterpenoid phytochemicals obtained from these two plants may serve as potential therapeutic agents.

## 1. Introduction

In the genomic era, genome-wide methylation profiles enable precise combinatorial drug screening for the treatment of various human diseases [1]. The one-drug, one-target mechanism is associated with various adverse effects, as it targets a single node in the complex molecular networks. Although moving toward combinatorial drug therapy involves another complex layer, i.e., the selection of an effective drug ratio for specific diseases—a laborious and expensive task [2]. To date, most of the functional combinations of drugs have been identified either from clinical trials or via prescription in medical practices. Similar approaches have been used in traditional medical practices since ancient times, but they have been associated with adverse reactions as well [3].

With the help of recent advancements, we can formulate drugs that exhibit reduced toxicity and can help people gain access to low cost medicines for various diseases. However, as most traditional medicine formulations prepared using regional plants are documented in the local language, the understanding of the mechanism of action of certain regional phytochemical products is further complicated by the language barrier. Thus, to identify a molecular signature that can be universally understood, we initiated genome-wide differential methylation screening for two oriental medicinal plant extracts (i.e., *Phlomis umbrosa* (PU) [4] and *Dipsacus asperoides* (DA) [5]), which were used to treat rats with mono-iodoacetate (MIA)-induced osteoarthritis (OA).

OA is a heterogeneous disease caused by various unknown factors [6] and is characterized by chronic joint pain caused by destruction of the cartilage and synovial membrane tissue at the knee joint. Extensive efforts have been made to understand the disease pathogenicity and to identify biomarkers for OA. For example, the insight of cell heterogeneity was assessed via a single-cell transcriptome study that identified the different clusters of synoviocytes (rich in the glycoproteins required for lubrication) and chondrocytes (which produce the structural components of cartilage) from the knee cartilage [7].

One of the main challenges in treating OA is the low-grade inflammation, which causes the destruction of the smooth tissues around the joints [6]. Interestingly rheumatoid arthritics (RA) also has the same phenotype; however, the underlying mechanisms are different from those involved in OA. The repurposing of RA drugs for OA failed due to this very reason [6]. To date, none of the approved drugs can reverse the phenotypes associated with OA and other bone-related diseases. Only a few molecules that target signaling pathways, such as Wnt and PI3K/AKT, are currently undergoing clinical trials for investigating their effects on OA [6,8]. Among these, some steroidal drugs are structurally similar to di- and tri-terpenoids [6].

*Phlomis umbrosa* Turczanimow (Family, Lamiaceae), is a medicinal plant, which has been used to treat allergic conditions [9], improve bone growth [10], and manage bone-related diseases, such as OA and osteoporosis [11,12]. Another medicinal plant, *Dipsacus asperoides* C.Y.Cheng & T.M.Ai (Family, Caprifoliaceae) is used to treat broken bones and liver abnormalities [13]. Both these medicinal plants are rich in iridoids, polyphenols, and saponins [4]. All three major groups of phytochemicals possess versatile therapeutic properties, which are being explored by scientists [14,15,16].

Particularly, iridoids are natural bioactive components that exert anti-inflammatory, hepatoprotective, neuroprotective, anti-tumor, and hypolipidemic effects [14]. For instance, loganin is an iridoid present in certain plants that exhibits a neuroprotective effect via the insulin-like growth factor-1 receptor (IGF1R) and glucagon-like peptide-1 receptor (GLP1R). It is also used in combination with LY294002 (PI3K/AKT inhibitor) to reduce extracellular matrix degradation in chondrocytes [17]. These two plant extracts were able to reduce an experimental OA phenotype in rat models, and nearly normalized the condition of the rats [4,5].

However, the molecular mechanisms and signaling pathways involved when cells are subjected to chemical perturbations are not clearly understood. Certain facts are known, including that methylation changes mostly occur during development and aging, and relatively few methylation changes are observed upon exposure to environmental stress factors. Considering data from several genome-wide methylation studies, we noted that chemical perturbations were observed in only five to ten percent of differentially methylated regions [18]. Taking these factors into consideration, we studied the differentially methylated regions (DMRs) in rats with MIA-induced OA treated with the above mentioned plant extracts. Additionally, the DMRs were correlated with molecular entities curated on DrugBank and the human genome to support this experimentalist approach.

## 2. Results

### 2.1. CpG Profiles and DMRs

**Figure 1:**
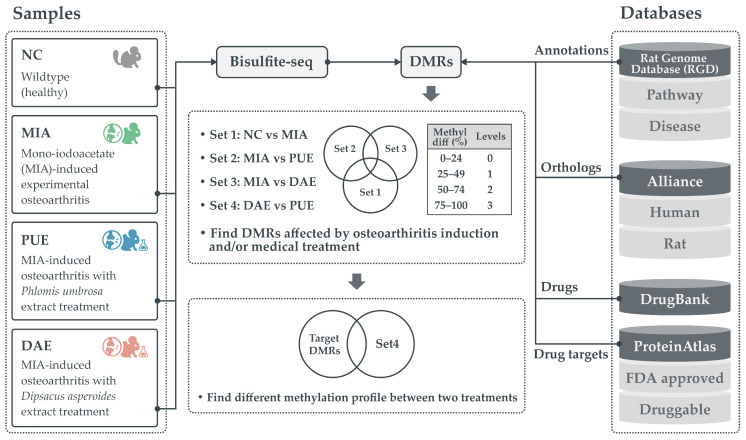
The detailed workflow employed in this study to identify the differentially regulated candidates in conditions corresponding to independent treatment with the two medicinal plant extracts.

**Figure 2:**
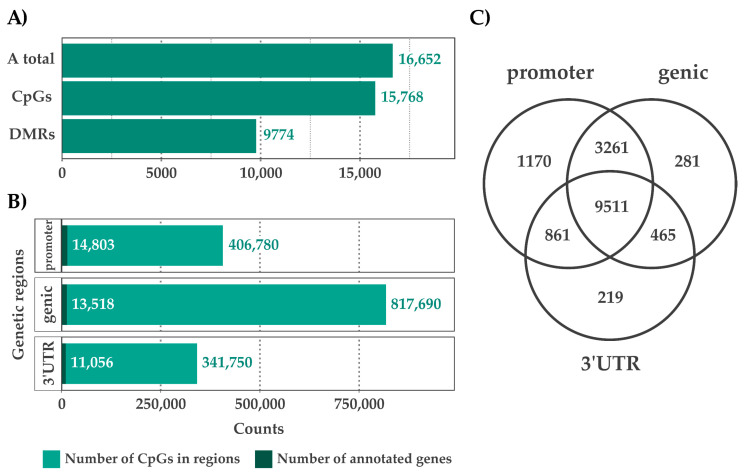
Summaries of the methylated regions. (**A**) Genes containing methylated regions from the total to differential methylated regions (DMRs). (**B**) Total CpGs and genic regions. (**C**) Distribution of CpG regions among the genetic regions.

The model includes three groups with three biological replicates each and a control (Figure 1). On average, 5.8 Gb of bases for each sample were sequenced, and 74.8% of the sequenced bases were mapped to the reference genome with 5.5 log2 coverage (Figure S1A,B). The principal component analysis and dendrogram cluster of CpG methylation sites clearly revealed the variance in the biological replicates (Figure S2A,B). In total, 1,861,526 CpG regions were covered—15,786 (94.8%) of the genes from the reference genome (Figure 2A), distributed across 15,090 (90.6%) promoters, 13,878 (92%) genic, and 11,143 (80.3%) 3-prime untranslated regions (3′-UTR) (Figure 2B)—and genes containing the CpG regions from all three region combinations are shown in a Venn-diagram in Figure 2C.

**Figure 3:**
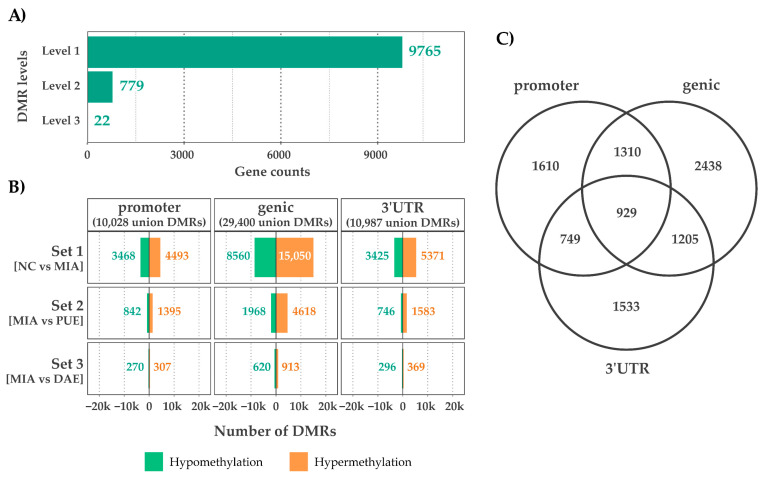
Summaries of differentially methylated regions. (**A**) Genes containing DMRs (level one to three). (**B**) DMRs in genic regions and combinations. (**C**) Distributions of hyper and hypomethylated regions across the genetic regions.

Approximately, 9511 (57.1%) genes contained CpGs in all three annotated regions. To obtain clear numbers in DMRs, we divided the DMRs into three levels as shown in Figure 1. In total, 9765 (58.6%) of the genes were shown to contain level 1 DMRs and only 801 (4.7%) contained level 2 and level 3 DMRs, which we consider the potential gene sub-set containing key markers for drug discovery (Figure 3A). The DMRs from all three annotated locations are presented as a Venn-diagram (Figure 3B), which shows that 929 genes contained CpGs from all three regions. With respect to CpGs, i.e., 50,415 (2.7%) were DMRs, and among those, 10,028, 29,400, and 10,987 DMRs were observed in the promoter, genic, and 3′-untranslated regions, respectively (Figure 3C).

### 2.2. DMRs Common between Plant Treatments and Experimentally-Induced OA

**Figure 4:**
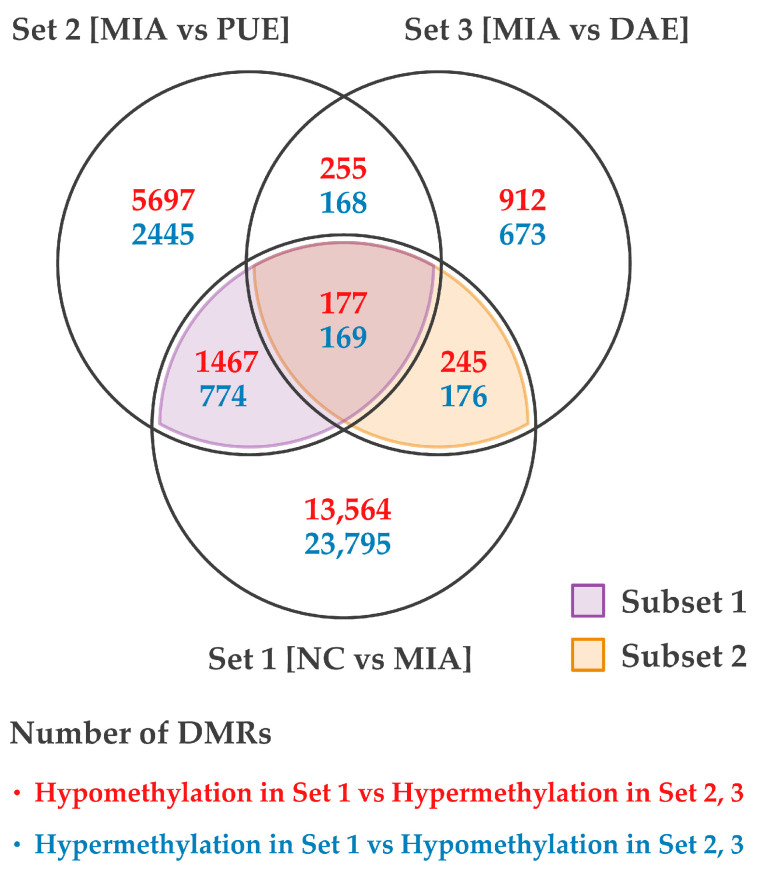
Summaries of the hyper- and hypo-methylated genes in subset 1 and 2.

To identify genes whose expression is regulated during MIA-induced disease onset and the subsequent treatment with medicinal plant extracts, we divided the dataset into four subsets, i.e., Set 1: OA (control vs MIA); Set 2: *Phlomis umbrosa* extract-treated (PUE); Set 3: *Dipsacus asperoides* extract-treated (DAE); and Set 4: the difference between PUE and DAE (Figure 1). We plotted DMRs in set one to three as a Venn-diagram (Figure 4).

Here, we considered that genes that overlapped between two gene sets i.e., a gene set containing genes that responded to chemical perturbation with MIA, which induced cartilage degradation and OA development, and a gene set that contained genes that responded to treatment with these medicinal plant extracts could hold the key to treating OA. These gene sets were termed subset 1 and subset 2 based on the plant extract against which a response was observed upon treatment in Figure 4 (subset 1, genes that responded to PUE; and subset 2, genes that responded to DAE).

Here, we selected the following genes: genes that exhibited level 2 expression and DMR signals in the regulatory the regions, i.e., promoter and 3′-UTR; and gene targets approved by the FDA to treat multiple diseases, for which some experimental evidence is available on DrugBank. In our analysis, *CKMT2*, *FDXR*, *FGR*, *GRIN2C*, *LGALS1*, *PCK1*, *PDXP*, and *THTPA* responded to both plant extracts. Further, *OGFOD1* exhibited a DMR signal in response to DAE treatment, whereas *SLC7A5*, *SOAT2*, *TAGLN2*, and *TUBD1* exhibited a DMR signal in response to PUE treatment. All combinations of genes are presented in Supplementary File S1.

### 2.3. Functional Annotation of CpGs

A systematic bioinformatic analysis was performed to simplify the target selection protocol in the subsequent experiments. Candidate molecules that have been proposed to be serve as drug targets were selected for use in animal experiments. The following public databases were used: DrugBank, to identify drugs that target the selected molecules; and the human proteome atlas database, to obtain information regarding FDA-approved and proposed targets to understand the importance of the target selection. Rat and human gene orthologs were identified using the Alliance database, and OA-related disease and pathway annotations were obtained from the Rat Genome Database (RGD) as described in the methods section.

**Figure 5:**
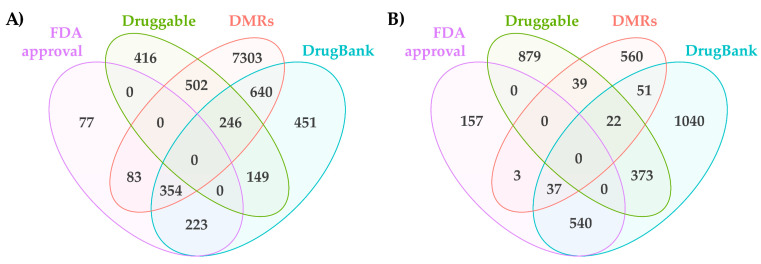
Summaries of DMRs overlapped with other public databases. (**A**) Level 1 DMR annotated genes and drug targeted genes (**B**) Level 2 and 3 DMR annotated genes and drug targeted genes.

**Figure 6:**
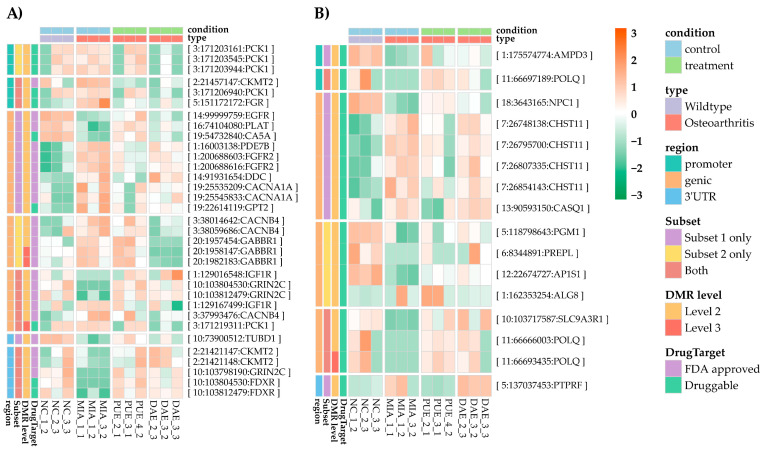
Level 1 and level 2 DMRs in subset 1 and 2 based on our data and the data hosted in public databases. (**A**) Genes in DrugBank. (**B**) Genes proposed as druggable targets.

Finally, a DMR network was established using all this information and is presented in Supplementary File S1. A combination of annotations is presented in Figure 5A,B, and our results showed that 151 genes contained level 2 DMRs and were connected with other three databases. Among these, 49 genes belonged to subset 1 and subset 2. Among these, drug information was present for 33 genes on DrugBank (Figure 6A), and 16 genes encoded proteins that are proposed to be druggable according to the Human Protein Atlas (Figure 6B). These genes could serve as effective targets to conduct further experiments aimed at investigating the effects of these two medicinal plant extracts or specific components identified in the extracts in the context of OA treatment (Table S1).

Among the genes in the two subsets, a few were notable, i.e., the gene encoding insulin-like growth factor-1 receptor (IGF1R), which has been shown to be targeted by loganin (an iridiod present in DAE) during the treatment of neurotoxicity in previous studies (Table S1), and *CHST11*, recently identified as an osteo-chondrodysplasia marker in a large scale genomic study [19,20], which has also been proposed as a druggable candidate (Figure 6B). These targets can be isolated from data analyzing the effect of these plant extracts in organisms.

### 2.4. PI3K/AKT Signaling Pathway

**Figure 7:**
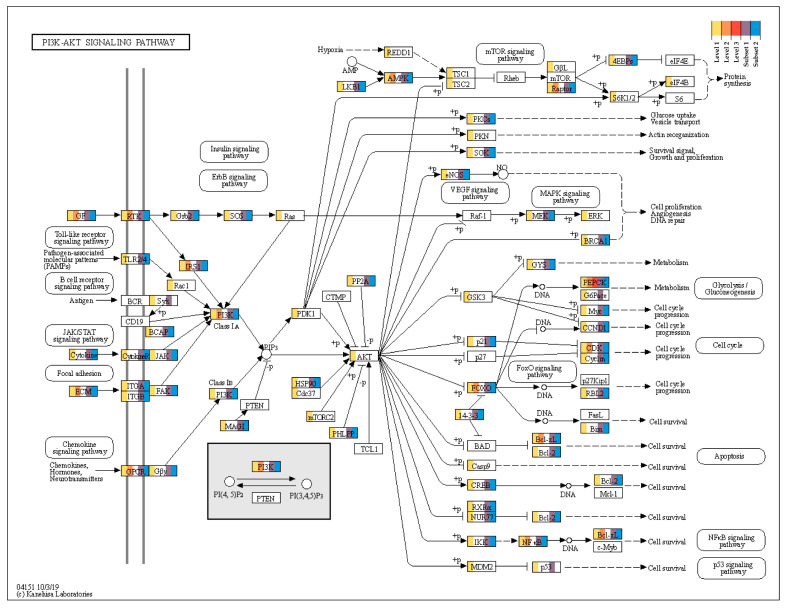
The PI3K/AKT signaling pathway with the heat map representation of level 1–3 and subset 1 and 2 DMRs (including all genic regions).

To understand how these two medicinal plants extracts regulated the signaling networks in OA, the level 2 DMRs were subjected to KEGG pathway enrichment analysis, resulting in 26 enriched pathways (Table 1). Among these, the PI3K/AKT pathway was highly enriched, followed by the cancer pathways. The PI3K/AKT signaling pathways comprise the following: ECM-receptor signaling, insulin signaling, focal adhesion signaling, and AMPK signaling (Figure 7).

This result clearly shows that the four networks in the PI3K/AKT pathway are highly associated with disease and medicinal plant treatments. Among these, two networks, the EGF-EGFR-PI3K pathway (EGF→EGFR→PI3K→AKT), and the IGF1R-PI3K pathway (IGF1→IGF1R→PI3K→PIP3→AKT→MTOR) are responsible for protein synthesis. The other two networks, the EGF-EGFR-RAS-PI3K pathway (EGF→EGFR→GRB2→SOS→RAS→PI3K→PIP3→AKT) and the EGF-EGFR-PI3K-NFKB signaling pathway (EGF→EGFR→PI3K→PIP3→AKT→IKK→NFKBIA→NFKB) are responsible for cell survival.

In total, 29 genes in PI3K/AKT pathway were level 2 and subset 1 and 2. Among those, eight genes (*COL11A2*, *COL3A1*, *CHAD*, *LAMA4*, *COL1A1*, *COL4A3*, *LAMC1*, and *COL2A1*) were involved in ECM-receptor interaction and five genes (*EFNA5*, *KITLG*, *ANGPT4*, *VEGFA*, and *FGF2*) encoded growth factor receptors, and five genes (*EPHA2*, *FGFR2*, *IGF1R*, *FGFR3*, and *EGFR*) encoded macrophage colony-stimulating factor 1 (RTK) receptor. In addition to these genes, *PEPCK* is responsible for the cell metabolism, and its expression was highly regulated in all three sets. These genes also contain DMRs in the regulatory modules (Figure S4). The expression of these genes could be modulated to destroy or reform the cartilage and synovial membrane tissues around the knee joint.

## 3. Discussion

This study was performed to understand the “many drugs with many targets” principle (also known as polypharmacy) by using the PU and DA extracts to treat MIA-induced OA. The biological replicates of the samples in this study ensured the significance of the findings (observed DMRs in genes known to be associated with “osteo”-related diseases). To understand the detailed functions of genes, correlations were identified between the ontologies/functional terms downloaded from well-known databases and the genome-wide methylation profile datasets.

Our OA dataset could enable further research in this field by OA researchers. PU and DA are rich in iridoid glycosides, (Table S1) that have been studied for their effect on OA in two forms, i.e., loganin and Shahzhiside methylester [21]. The main advantage of iridoid glycosides in the treatment of OA is their hepatoprotective effect [13,14], especially considering the fact that the major factor limiting the use of natural extracts to treat the diseases is liver toxicity.

Similarly, the anti-inflammatory properties of other natural compounds, such as genipin (iridoid glycoside derivative), aucubin (iridoid glycoside), nuezhenelenoliciside (secoiridoid), tormentic acid (triterpenoid), ginsenosides (triterpenoids) [22], leonurine (alkaloid), vanillic acid (catechin-type phenol), and scoparone (natural organic component) have been investigated in both chondrocyte and cartilage injuries [8]. As our extracts also contain triterpenoids, DAE in particular contains Akebia saponin D, which exerts various therapeutic effects (Table S1 and Figure S5) [23] and was also used to reverse the corticosterone hypersecretion in a rat model of Alzheimer’s disease.

Saponins also exhibit functional activities in the context of disease signaling pathways [16]. Thus, saponins in DAE could serve as alternatives to steroid drugs, such as dexamethasone [24], 17β-estradiol [6], and corticosteroids [6], which are widely used to treat OA, thereby, reducing the adverse reactions associated with these immunosuppressive drugs (e.g., new-onset diabetes is an adverse reaction associated with glucocorticoid use [25]). Genipin, an iridoid glycoside can regulate glucose homeostasis via interactions with uncoupling protein 2 (UCP2) [26].

Another phenomena in OA is the loss of 17β-estradiol (a steroid hormone), a phenomenon associated with hip and knee pathogenesis in OA [27]. This phenotype can be managed by supplementation with saponin-rich extracts that have low liver toxicity and bioavailability [28]. The known bioactive compounds in these extracts are administered individually and in combination with other drugs. For example, when co-administered with LY294002 (PI3K/AKT inhibitor), loganin attenuates cartilage degeneration and bone sclerosis in subchondral bone.

Alternatively, OA drug discovery research also focuses on different molecular signaling patterns through the literature mining approach, which explains the signaling pathways and genes studied for OA up to 2018 [17]. The Osteoarthritis society also summarized the progress in disease management [29]. Furthermore, Tonia L Vincent explained the convergence of molecular signaling in the in-vitro model and humans and the progress of OA drug discovery up to 2020 [6]. All these studies summarized that OA therapeutics were focused on targeting PI3K/AKT/mTOR signaling pathway [8], activating the polarized macrophages [30], growth factor therapies [6], and low-grade inflammation [29].

The iridoid components were also predicted to be a good natural component that promotes nerve growth and other blood vessel growth [14]. Our result strongly correlates with their suggestions, as DMR genes were enriched in the PI3K/AKT signaling pathway and sub-networks as depicted in Section 2.4. Genes involved in the extra cellular matrix (ECM)–receptor interactions could be therapeutic targets because the extra cellular matrix (ECM) is an important layer of most of the tissues in our body, and it anchors hundreds of proteins to maintain the structural flexibility of the tissues, particularly in the joint cartilage [31].

ECM is also known as the key modulator that frequently responds to external stimuli and makes the decision regarding the cell’s fate by altering the intracellular signaling pathways [32]. Through mathematical modeling, Jordan F Hastings proposed ECM as a key regulatory sub-network that could decide the cell responses, behavior, phenotype, and drug response for acute and chronic diseases [32]. ECM components can act as ligands to activate signaling networks in both healthy and diseased states. ECM components, such as laminin, and collagen are used to activate the integrin family receptors.

In our results, the collagens (COL11A2, COL3A1, COL1A1, COL4A3, and COL2A1) and laminin (LAMA4, and LAMC1) were highly regulated to ensure these phenomena. Focal adhesion kinase (FAK)—involved in cell adhesion dynamics and mobility via the ECM—activates the PI3K/AKT signaling pathways, which has the key to regulate the proliferation, progression, metabolism, and survival mechanisms. This is a potential signaling mechanism known for wound healing and tissue repair process [8]. In the case of osteoarthritic drug discovery, the ECM degradation and formation mechanism has also gained attention [33].

The expression of growth factor receptors, such as *KITLG* [34], *ANGPT4* [35], *VEGFA* [36], and *FGF2* [37], are regulated by the 17β-estradiol. Similarly, our results also showed DMRs in these growth factors. Therefore, we hypothesize that Akebia saponin D could directly influence these growth factors to treat OA with these plant extracts. These can be further validated in detailed molecular experiments, and the genes, such as *EPHA2*, *FGFR2*, *IGF1R*, *FGFR3*, and *EGFR*, can be used to trigger polarized macrophages via the macrophage colony-stimulating factor 1 (RTK) receptor since these receptors are highly regulated in our dataset.

Another mode of targeting OA involves therapeutics via altering low-grade inflammation, which can be achieved by conducting a detailed research of the iridoids for AKT signaling via the above receptors. A study reported that the IGF1R is a strong activator of AKT phosphorylation and can activate AKT to promote synthesis of collagen II [14]. To the best of our knowledge, this is the first methylome profiled dataset for these two plant extracts that are used to treat experimental OA. This study provides detailed insight regarding potential OA therapeutic agents from these two plant extracts and specific chemical components.

## 4. Materials and Methods

### 4.1. Ethics Statement

Fifteen male Sprague–Dawley rats (7 weeks old) were purchased from Daehan Bio Link, Inc. (Eumseong, Chungcheongbuk-do, Korea). The animal experiment procedures were performed by following the guidelines of the Institutional Animal Care and Use Committee and were approved by the Ethical Committee of Kyungpook National University (NO. KNU 2018-0091). While conducting this study, all efforts were made to maximize the scientific benefit while minimizing the suffering of the animals.

### 4.2. Sample Collection and Experimental Design

*Dipsacus asperoides* C.Y.Cheng & T.M.Ai (DA) and *Phlomis umbrosa* Turczanimow (PU) were purchased from Naemome Dah Herbal Medicine (Ulsan Metropolitan, Korea) and MyRyeung Herbal Medicine (Pocheon-si, Gyeonggi-do, Korea), respectively. Two species were used to carry out morphological analysis by Dr. Goya Choi and genetic analysis by Dr. Byeong Cheol Moon of Korea Institute of Oriental Medicine. Two voucher specimens (DAE; No. 2-17-0059~2-17-0060, PUE; No. 2-17-0072) were deposited in the Korean Herbarium of Standard Herbal. The medicinal plant extracts from PU and from DA were acquired as explained in the previous study on transcriptome profiling [4,5].

The experimental design and the rats in this study were also brought from a previous study, while using one additional group of DAE (MIA-injected with saline and DAE) rats. A total of four groups (*n* = 3 per group), including untreated with saline (NC: normal control), MIA-injected with saline (MIA), MIA + DAE treatment (DAE), and MIA + PUE treatment (PUE), of rats were used in this study. To induce osteoarthritis (OA) in rats, MIA (3 mg/50 μL saline) was directly injected into the intra-articular space of the right knee of each rat while they were subjected to inhalation anesthesia. The medicinal plant extract treatments (PUE and DAE) were dosed daily at 200 mg/kg body weight for 21 days.

### 4.3. Library Preparation and Sequencing

The total DNA for bisulfite-seq was collected in the rat samples. Each rat’s cartilage was collected, and the DNA was extracted using a QuickGene DNA tissue kit, following the manufacturer’s protocol and was immediately frozen in liquid nitrogen and preserved for further use. The DNA quantification was carried out as a quality check, and three samples in each group were selected for library construction and sequencing. Bisulfite-seq libraries for sequencing were prepared using the SureSelect Methyl-Seq Library Prep Kit (Agilent Technologies, Santa Clara, CA, USA). The constructed libraries were sequenced using a Novaseq 6000 Sequencing System (Illumina, San Diego, CA, USA), yielding more than 2 × 50 million reads with 2 × 100 base-pair (bp) read lengths for 12 samples.

### 4.4. Genome Wide Methylation Patterns from Bisulfite-Seq Data

The reads were trimmed using trimmomatic (v0.38) after a quality check to remove low quality reads and adapters after sequencing. The bisulfite-seq reads were mapped to the rat DNA reference genome (rn6) acquired from NCBI using Bowtie2 (v2.3.4) via Bismark (v0.20.0) [38] and sorted and de-duplicated using samtools (v1.9) via Bismark. The read process statistics of the bisulfite-seq data were detailed in Figure S1. After the alignments, the read coverages on the cytosine sites were extracted using Bismark.

The coverage files were used to identify DMRs site by site using the methylKit R package (v1.10.0) [39]. The differential methylation of hyper- or hypo-methylated regions was calculated with a 25% methylation difference between groups, and differences were considered statistically significant at *p*-values < 0.05. The DMRs (for CpG, CHH, and CHG sites) were then annotated for genomic features (promoter, genic, and 3′UTR). All the DMRs were divided into three levels as shown in Figure 1 (Level 1: 25–49%, Level 2: 50–74%, and Level 3: 75–100%).

### 4.5. Gene Set Enrichment Analysis among Union Genes in DMRs

The methylation profile heatmap of union genes in the DMRs was prepared with the pheatmap R package (v1.0.12) and hierarchically clustered with the Euclidean distance and ward.D clustering algorithm. To identify the function and pathways of the clustered gene set, both datasets were evaluated using PANTHER [40] and clusterProfiler (v3.12.0) [41] in R using the gene ontology enrichment functions and org.Rn.eg.db library (v3.8.2) to build Gene Ontology (GO) terms.

The genes in each heatmap cluster were grouped into various categories involved in similar functions (biological processes, molecular functions, and cellular components) and pathways through the GO database and Kyoto Encyclopedia of Genes and Genomes pathway (KEGG) database to reveal their functional roles. The statistical enrichment of GO terms for the genes was tested using Fisher’s exact test with an adjusted *p*-value of 0.05. Finally, the KEGG enrichment pathway and GO analysis for DMRs (Level 2) was conducted with the DAVID online functional analysis tool (https://david.ncifcrf.gov/).

### 4.6. Functional Database

As shown in Figure 1, functional annotations for each gene were made as per the drug discovery protocol. Four datasets are included in Supplementary File S1, i.e., the DrugBank [42], Alliance [43], Human Protein Atlas [44], and Rat Genome Database (RGD) [45]. Here, we include the DrugBank ID for each gene, to easily navigate the details regarding known drugs from DrugBank from the complete database xml file. Second is the Alliance database from the files DISEASE-ALLIANCE_RGD_37.tsv, and ORTHOLOGY-ALLIANCE_COMBINED_37.tsv. Third is the RGD database to obtain the detailed information of diseases and pathway annotations from the files rattus_terms_pw and rattus_terms_rdo. Fourth, the FDA approved, potential drug candidates were downloaded from the human protein atlas database.

## Figures and Tables

**Table 1:** Enriched KEGG pathways for DMRs in level 2–3 genes.

Term	Count	%	*p*-Value	Genes	Fold Enrichment	FDR
rno04151:PI3K/AKT signaling pathway	29	3.70	0.00	Vegfa, Col3a1, Angpt4, Lamc1, Nfkb1, Col2a1, Prkaa1, Col11a2, Kitlg, Col4a3, Irs1, Col1a1, Rptor, Foxo3, Egfr, Bcl2l1, Fgfr3, Fgf2, Igf1r, Lama4, Cdk6, Efna5, Chad, Fgfr2, Pck1, Epha2, Lpar3, Pik3r2, Jak1	2.08	0.08
rno05200:Pathways in cancer	28	3.58	0.01	Vegfa, Lamc1, Nfkb1, Kitlg, Col4a3, Rxrb, Egfr, Mecom, Bcl2l1, Fgfr3, Fgf2, Igf1r, Wnt4, Ptch1, Lama4, Axin2, Fzd9, Cdk6, Ctnnb1, Prkcg, Ppard, Gna13, Fgfr2, Hif1a, Lpar3, Pik3r2, Jak1, Brca2	1.72	0.12
rno04144:Endocytosis	22	2.81	0.00	Pdcd6ip, Smurf1, Pard3, Chmp4c, Mvb12b, RT1-M6-2, Git2, Chmp1b, Dnm1, Smurf2, Rab11fip4, Acap1, Fgfr2, Egfr, Nedd4l, Rab31, Dnajc6, Zfyve27, Fgfr3, RT1-T24-3, Igf1r, Wipf1	1.99	0.12
rno04014:Ras signaling pathway	21	2.68	0.00	Rasgrf2, Vegfa, Angpt4, Rgl1, Nfkb1, Shc3, Prkcg, Gab1, Efna5, Kitlg, Fgfr2, Egfr, Bcl2l1, Shc4, Pla2g12a, Fgfr3, Fgf2, Epha2, Rasa3, Pik3r2, Igf1r	2.22	0.12
rno05205:Proteoglycans in cancer	18	2.30	0.00	Vegfa, Fzd9, Ank3, Hcls1, Ctnnb1, Prkcg, Gab1, Itpr2, Hbegf, Hif1a, Egfr, Fgf2, Pik3r2, Igf1r, Ptch1, Wnt4, Gpc3, Cd44	2.18	0.12
rno04510:Focal adhesion	18	2.30	0.01	Vegfa, Col3a1, Lamc1, Ctnnb1, Shc3, Col2a1, Prkcg, Col11a2, Parva, Col4a3, Chad, Col1a1, Dock1, Egfr, Shc4, Pik3r2, Igf1r, Lama4	2.09	0.12
rno04015:Rap1 signaling pathway	17	2.17	0.02	Pard3, Vegfa, Angpt4, Ctnnb1, Prkcg, Efna5, Kitlg, Adora2b, Fgfr2, Egfr, Fgfr3, Gnao1, Fgf2, Epha2, Lpar3, Pik3r2, Igf1r	1.92	0.23
rno04020:Calcium signaling pathway	16	2.04	0.01	Sphk2, Prkcg, Adra1b, Itpr2, Ppp3ca, Nos1, Adora2b, Orai1, Itpkb, Egfr, Cacna1a, Grin2c, Atp2b1, Vdac3, Gnal, Ptk2b	2.11	0.15
rno04910:Insulin signaling pathway	14	1.79	0.00	Prkab1, Shc3, Prkaa1, Ptprf, Irs1, Ppp1r3c, Rptor, Hk2, Prkab2, Pck1, Shc4, Rhoq, Pik3r2, Acacb	2.44	0.12
rno05206:MicroRNAs in cancer	14	1.79	0.01	Vegfa, Cdk6, Nfkb1, Prkcg, Irs1, Slc7a1, Prkce, Rptor, Egfr, Shc4, Reck, Fgfr3, Trim71, Cd44	2.39	0.12
rno04152:AMPK signaling pathway	13	1.66	0.01	Scd2, Prkab1, Prkaa1, Lepr, Pfkfb3, Irs1, Rptor, Foxo3, Prkab2, Pck1, Pik3r2, Acacb, Igf1r	2.47	0.12
rno04932:Non-alcoholic fatty liver disease (NAFLD)	13	1.66	0.03	Ndufa4l2, Cox4i2, Prkab1, Nfkb1, Prkaa1, Lepr, Ndufs7, Irs1, Prkab2, Casp7, Ndufb8, Ndufs8, Pik3r2	1.96	0.41
rno04931:Insulin resistance	12	1.53	0.01	Irs1, Prkce, Ppp1r3c, Prkab2, Prkab1, Nfkb1, Pck1, Prkaa1, Ptprf, Rps6ka2, Pik3r2, Acacb	2.66	0.12
rno04512:ECM-receptor interaction	11	1.40	0.00	Col3a1, Col1a1, Chad, Lamc1, Col2a1, Col11a2, Gp9, Col4a3, Cd47, Lama4, Cd44	3.01	0.12
rno04974:Protein digestion and absorption	11	1.40	0.00	Col3a1, Col1a1, Kcnk5, Col2a1, Col11a2, Atp1a4, Col4a3, Slc7a8, Kcnq1, Col17a1, Eln	3.01	0.12
rno04066:HIF-1 signaling pathway	11	1.40	0.01	Vegfa, Serpine1, Hk2, Hif1a, Angpt4, Egfr, Nfkb1, Prkcg, Pfkfb3, Pik3r2, Igf1r	2.63	0.15
rno05146:Amoebiasis	11	1.40	0.02	Col3a1, Col1a1, Lamc1, Nfkb1, Col2a1, Prkcg, Col11a2, Col4a3, Pik3r2, Gnal, Lama4	2.39	0.23
rno04920:Adipocytokine signaling pathway	10	1.28	0.00	Irs1, Rxrb, Prkab2, Prkab1, Nfkb1, Pck1, Prkaa1, Lepr, Acacb, Nfkbib	3.25	0.12
rno05100:Bacterial invasion of epithelial cells	9	1.15	0.02	Dock1, Hcls1, Ctnnb1, Shc3, Septin8, Shc4, Gab1, Dnm1, Pik3r2	2.71	0.24
rno04915:Estrogen signaling pathway	9	1.15	0.04	Hbegf, Fkbp5, Egfr, Shc3, Shc4, Gabbr1, Gnao1, Itpr2, Pik3r2	2.28	0.48
rno05212:Pancreatic cancer	8	1.02	0.02	Vegfa, Egfr, Cdk6, Bcl2l1, Nfkb1, Jak1, Pik3r2, Brca2	3.05	0.23
rno04520:Adherens junction	8	1.02	0.03	Pard3, Ssx2ip, Lmo7, Egfr, Ctnnb1, Ptprf, Ptprm, Igf1r	2.67	0.38
rno04730:Long-term depression	7	0.89	0.04	Gna13, Cacna1a, Prkcg, Gnao1, Itpr2, Nos1, Igf1r	2.75	0.48
rno05230:Central carbon metabolism in cancer	7	0.89	0.05	Fgfr2, Hk2, Hif1a, Slc7a5, Egfr, Fgfr3, Pik3r2	2.67	0.48
rno05214:Glioma	7	0.89	0.05	Egfr, Cdk6, Shc3, Shc4, Prkcg, Pik3r2, Igf1r	2.62	0.49
rno00220:Arginine biosynthesis	4	0.51	0.05	Got2, Acy1, Gpt2, Nos1	4.87	0.48

## Data Availability

The complete sequences generated in this study were deposited in the SRA repository under the accession PRJNA717328. The metadata of the individual samples were given in Supplementary File S1.

## Supplementary Materials

The following are available online at https://www.mdpi.com/article/10.3390/plants10061132/s1, Figure S1: Reference mapping summaries A: preprocessing and mapping statistics; B: coverage.; Figure S2: Sample grouping A: PCA with CpG sites.; B: Dendrogram with CpG methylated sites.; Figure S3: Set4 A: DMRs and B: hyper and hypo methylated genes and subsets overlapped summaries.; Figure S4: PI3K-AKT signaling pathway with the heat map representation of DMRs (only with promoter and 3’UTR regions) Level 1-3 and subset one and two; Figure S5: The chemical structure of Akebia saponin D.; Table S1: List of bioactive chemical components present in the medicinal plant extracts.
