# Supplementary material for: Genome-Wide Differential Methylation Profiles from Two Terpene-Rich Medicinal Plant Extracts Administered in Osteoarthritis Rats
Source: Plants (Basel). 2021 Jun 2;10(6):1132. doi: 10.3390/plants10061132 (PMC8227118; doi:10.3390/plants10061132)
Supplement: Supplementary file 1 [file plants-10-01132-s001.zip › Supplementary_Figures and Tables.pdf]

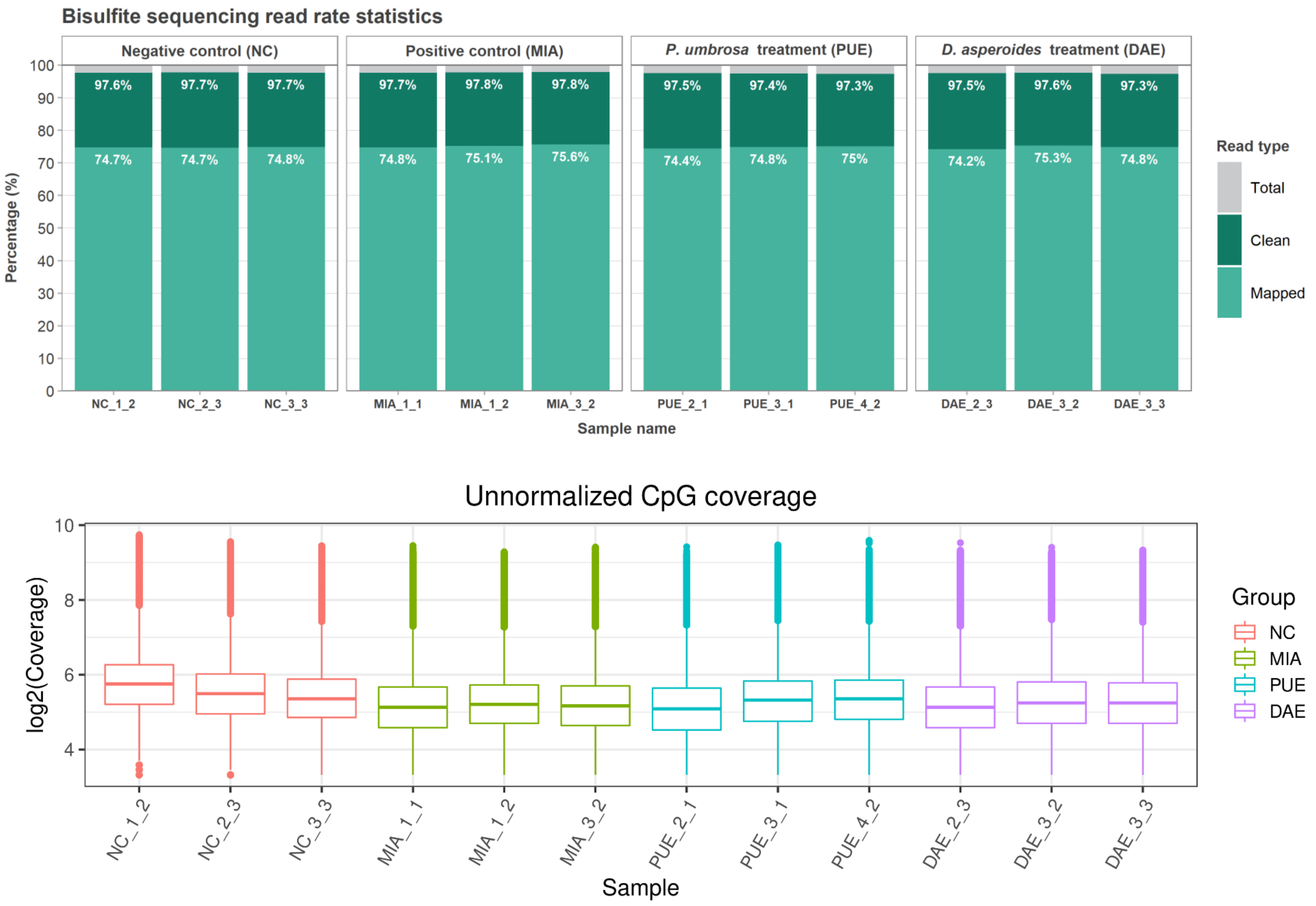

Figure S1: Reference mapping summaries A: preprocessing and mapping statistics; B: coverage statistics.

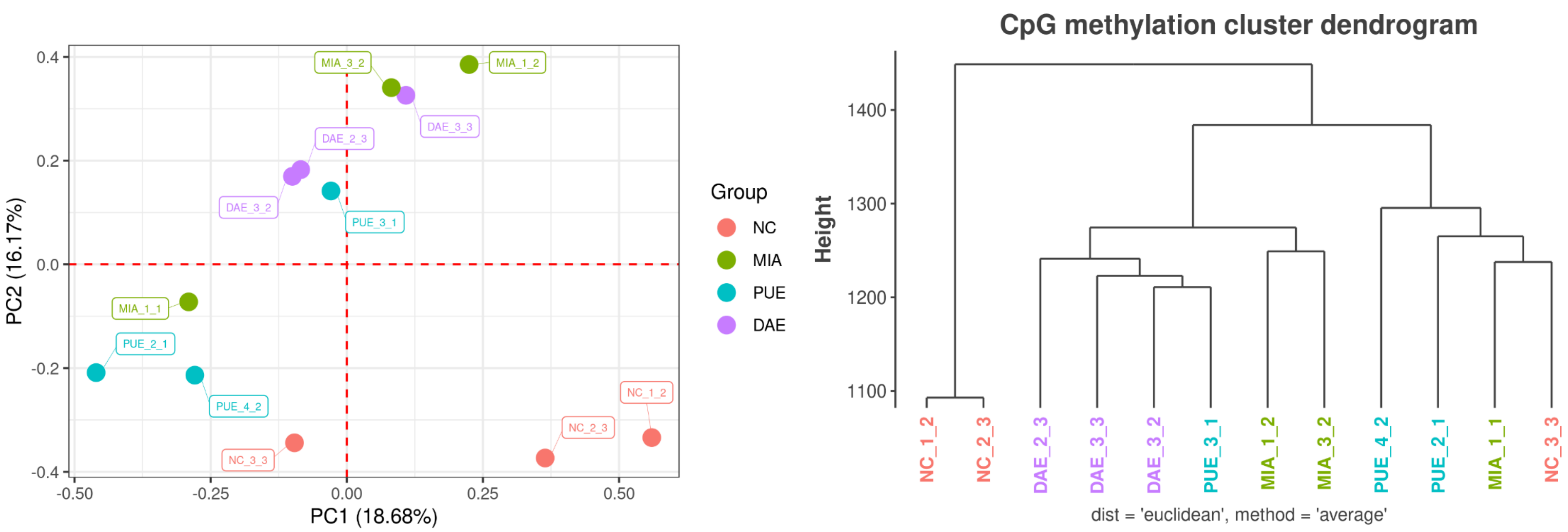

Figure S2: Sample grouping A: PCA with CpG sites ; B: Dendrogram with CpG methylated sites.

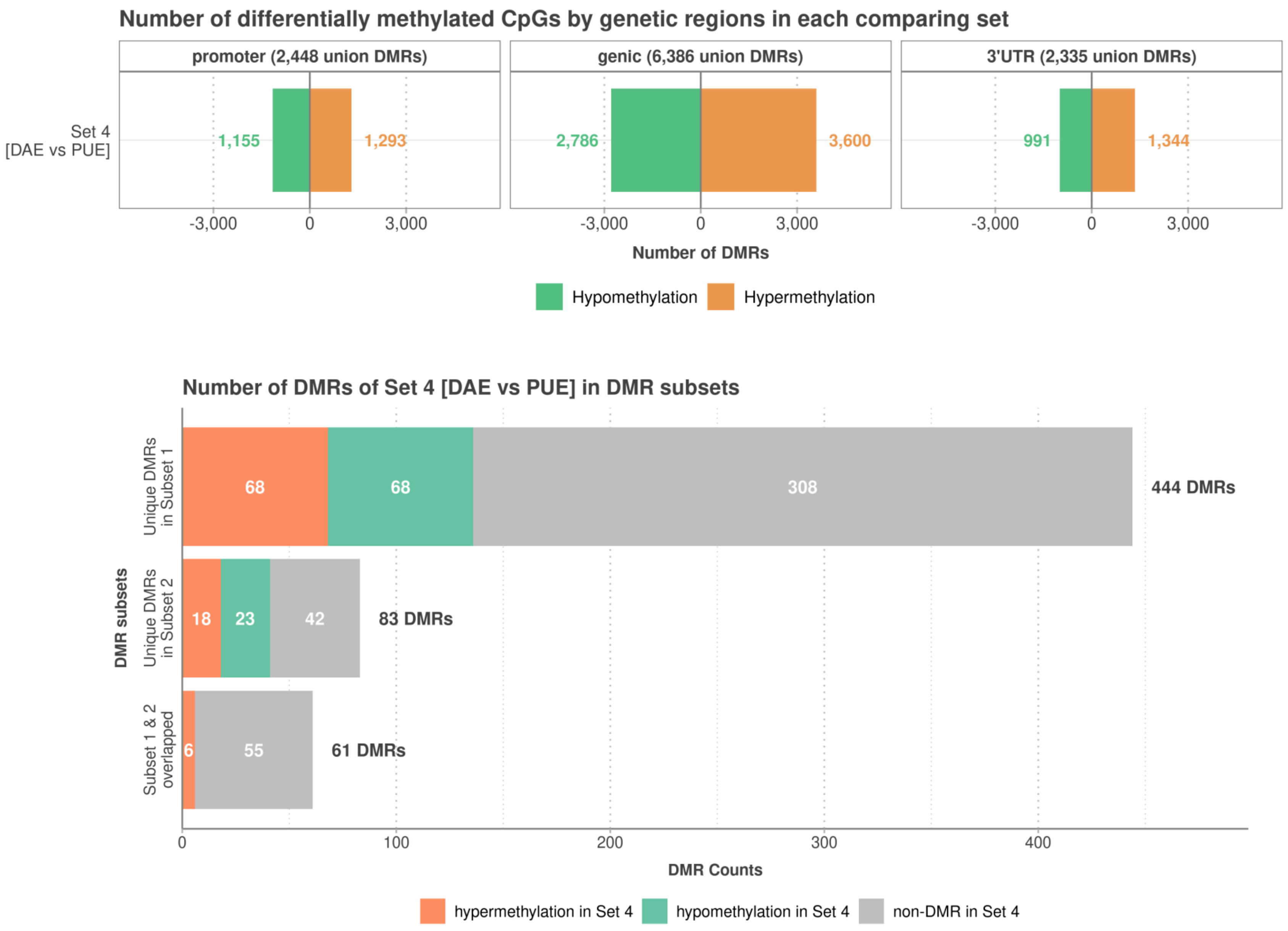

Figure S3: Set4 A: DMRs and B: hyper and hypo methylated genes and subsets overlapped summaries

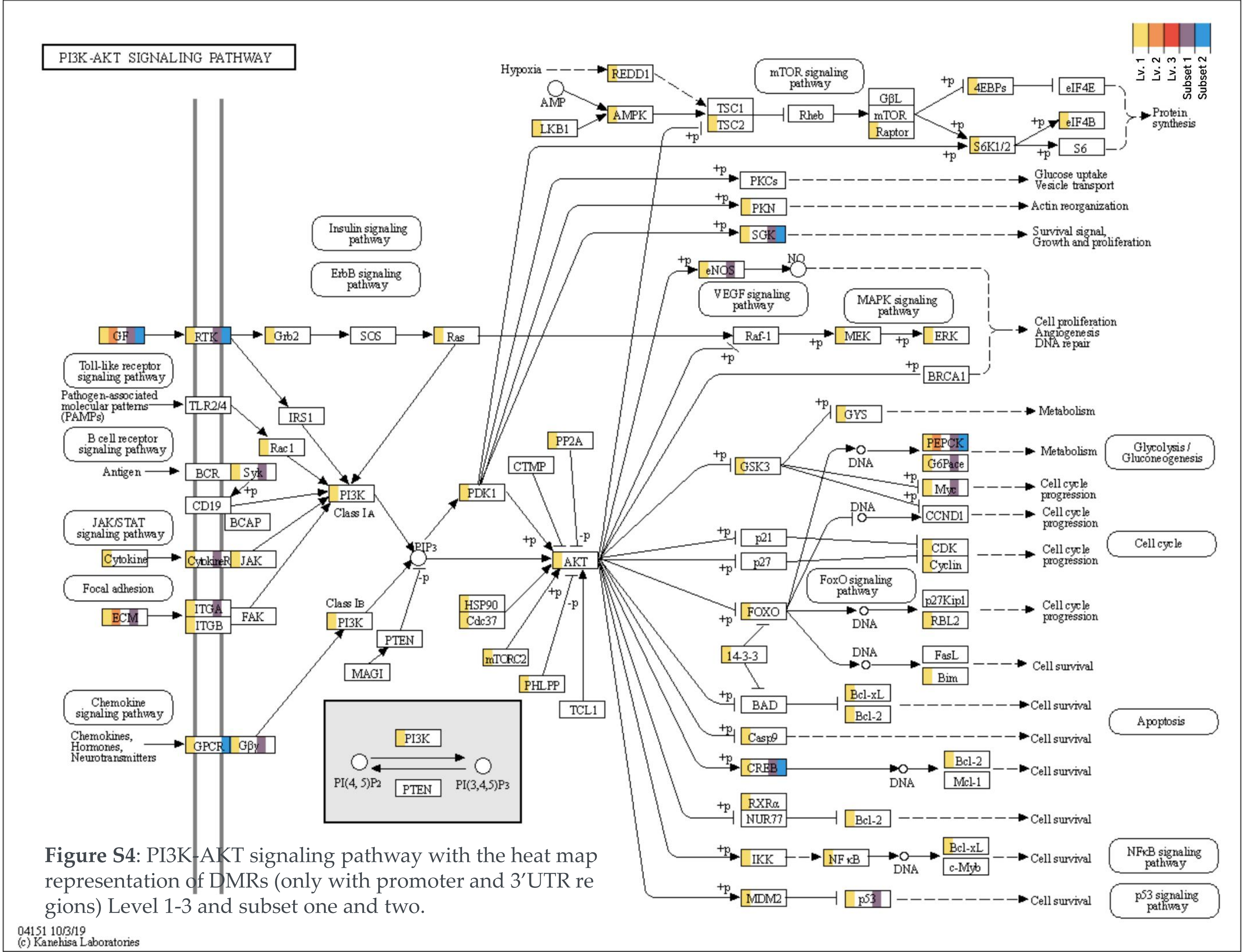

Figure S4: PI3K-AKT signaling pathway with the heat map representation of DMRs (only with promoter and 3'UTR regions) Level 1-3 and subset one and two.

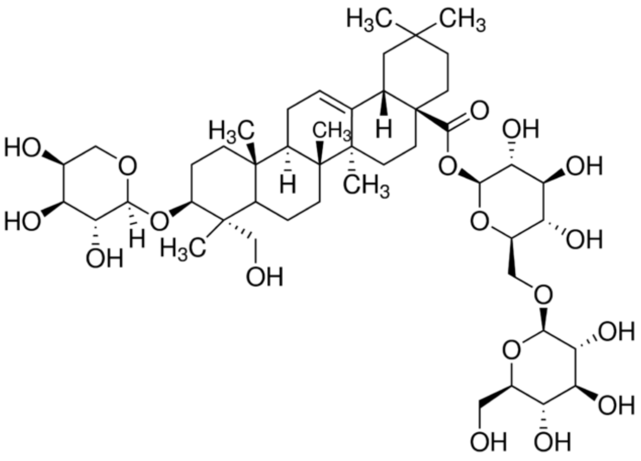

Figure S5: The chemical structure of Akebia saponin D

Bioactive Chemical Class	*Phlomis umbrosa*	*Dipsacus asperoides*
Iridoid glucosides (monoterpenoids)	Umbroside<br>Shahzhiside methylester<br>Sesamoside	Loganin<br>Loganic acid
Secoiridioids (monoterpenoids)	-	Sweroside
Phenols	-	Chlorogenic acid<br>Isochlorogenic acid A
Saponins (Triterpenoids)	-	Akebia saponin D

Table S1: List of bioactive chemical components present in the medicinal plant extracts
